# Protocol for optimizing the utilization rates of coronary angiography in Norway

**DOI:** 10.1007/s43999-026-00096-2

**Published:** 2026-07-30

**Authors:** Andreas Kristensen, Marit Herder, Erlend Aune, Signe Helene Forsdahl, Jon Olav Haugstvedt, Amjid Iqbal, Kristian Kolnes, Jon Bjarne Leiknes, Anagha P. Parkar, Svein Rotevatn, Christian Thoresen, Rune Wiseth, Ole Tjomsland

**Affiliations:** 1 The Arctic University of Norway, Tromsø, Norway; 2 Centre for Clinical Evaluation and Documentation (SKDE), Tromsø, Norway; 3 Vestfold Hospital Trust, Tønsberg, Norway; 4 University Hospital of North Norway, Tromsø, Norway; 5 Oslo University Hospital, Oslo, Norway; 6 Volda Hospital, Møre og Romsdal Hospital Trust, Volda, Norway; 7 Stavanger University Hospital, Stavanger, Norway; 8 Haraldsplass Deaconess Hospital, Bergen, Norway; 9 Haukeland University Hospital, Bergen, Norway; 10 South-Eastern Norway Regional Health Authority, Hamar, Norway; 11 Clinic of Cardiology, St Olavs University Hospital, Trondheim, Norway; 12 Faculty of Health Science, University of Stavanger, Stavanger, Norway

**Keywords:** Coronary angiography, Coronary CT angiography (CCTA), CAD-RADS, ESC guidelines, Norway

## Abstract

**Background:**

Invasive coronary angiography (CAG) remains widely used in Norway, with 37% of procedures in 2022 yielding normal findings, indicating potential overuse.

**Methods:**

A national multidisciplinary task force performed a retrospective registry-based analysis and reviewed the European Society of Cardiology (ESC) guidelines to identify strategies aimed at reducing the utilization of CAG in patients with suspected coronary disease.

**Results:**

Significant regional variation in CAG and Coronary Computed Tomography Angiography (CCTA) utilization was observed. Complication rates for CAG in stable patients were low, 0.6%. In patients with low and moderate pre-test probability and suspected Chronic Coronary Syndrome (CCS), CCTA is recommended as a first-line imaging test.

**Conclusion:**

Standardization of CCTA protocols and reports, using CAD-RADS 2.0, mandatory CCTA registration in Norwegian Registry of Invasive Cardiology (NORIC), and formal collaboration between radiologists and cardiologists are essential initiatives. The aim is to reduce invasive procedures not resulting in revascularization by 5–10 percentage points nationally.

## Introduction

Multiple counselling-based interventions aimed at reducing the use of low-value procedures, i.e. procedures that are medically unnecessary because they provide minimal or no net health benefit, have so far had only limited impact on utilisation rates [1, 2]. The underlying assumption in such initiatives is that increased knowledge, together with data demonstrating unwarranted variation, is sufficient to change clinical practice. Unfortunately, this has not been the case [3]. Numerous studies have documented complex organisational, professional, and patient-level barriers to change [4].

Norwegian Operational Group for Reassessment (The NOR Programme) is a Norwegian national initiative, launched by the Chief Executive Officers (CEOs) of the Norwegian Regional Health Authorities (RHAs) and governed by the Centre for Clinical Evaluation and Documentation (SKDE), to reduce the use of low-value procedures [5]. Criteria used to select initiatives were;

(i) International designation as low-value care e.g. Choosing Wisely [6], Evidence-Based Intervention Programme [7].
(ii) Moderate to high patient risk.
(iii) High cost.
(iv) High volume and geographical variation.

Based on these criteria the use of invasive coronary angiography (CAG) in patients with suspected or known chronic coronary syndrome (CCS) was selected as one of three initiatives.

Traditionally, suspected CCS has been assessed by exercise ECG and CAG to confirm diagnosis, grade coronary pathology, and treat obstructive ischaemia-inducing lesions with revascularisation (percutaneous coronary intervention (PCI) or coronary artery bypass grafting (CABG)). According to the NHS EBI Recommendations, exercise ECG has no role in screening for coronary heart disease in asymptomatic or low-risk adults, due to its poor diagnostic performance and low pre-test probability [8]. Recent evidence suggests that revascularisation in CCS rarely improves prognosis over optimal medical therapy alone [9, 10]. CAG is costly and carries procedural risks (e.g., bleeding, contrast-induced nephropathy, stroke, myocardial infarction, dissection, or death) [11]. Coronary CT angiography (CCTA), a non-invasive and generally well-tolerated modality, has emerged as the first-line investigation for individuals at known or suspected risk of coronary artery disease [12]. However, CAG with procedural risk and resource implications remains a cornerstone in the diagnosis of coronary artery disease. In Norway, approximately 30,000 CAG procedures are performed annually, with 37% yielding normal findings [13]. This short report summarizes the report from the NOR expert group with recommendations aimed at reducing the number of unnecessary invasive procedures. 

## Methods

A multidisciplinary task force comprising cardiologists and radiologists from all RHAs conducted eight structured meetings. Data used in the work was retrieved from Norwegian Registry of Invasive Cardiology (NORIC) and Senter for klinisk dokumentasjon og evaluering (Center for Clinical Documentation and Evaluation – SKDE). Informed recommendations were used to adjust the recommended clinical pathway for patients with suspected coronary syndrome. The recommendations were based on contemporary evidence on utilization of CCTA versus CAG combined with the 2024 ESC Guidelines for the management of chronic coronary syndromes [14], the 2023 ESC guidelines for the management of acute coronary syndromes [15], and the 2025 ESC guidelines for management of valvular heart disease [16]. The review focused mainly on elective CAG procedures and assessed regional variation, complication rates, and adherence to non-invasive diagnostic pathways.

## Results

In 2022, 16,640 diagnostic CAGs were performed in Norway without resulting in revascularization procedures (percutaneous coronary intervention (PCI) or coronary artery bypass grafting (CABG)). Of these, 0.6% resulted in major complications according to the NORIC registry, primarily access-site bleeding. Regional variation was substantial. The rate of CAGs showing no obstructive coronary disease ranged from 30% to 50% across hospitals. CCTA utilization varied fourfold between regions. ESC guidelines recommend CCTA as first-line imaging for patients with suspected coronary disease and a low to moderate pre-test probability (5–50%). Despite this, invasive strategies remain prevalent.

## Discussion

Norway is a geographically large and elongated country with a dispersed population of 5.3 million. Coronary angiography (CAG) procedures are centralised to eight hospitals.

The findings in our study underscore the need for systematic changes in coronary diagnostic pathways. Key recommendations include:

1. Prioritizing CCTA in patients with stable chest pain and structural heart disease
2. Implementing standardized CCTA protocols and reports using CAD-RADS 2.0 [17]
3. Establishing mandatory CCTA registration in NORIC
4. Fostering collaboration between radiology and cardiology departments, including private providers.

Through these measures a 5–10 percentage point reduction of CAGs not resulting in revascularization is considered achievable. 

### Limitations

The interventions in this protocol are implemented within Norway’s public health system, where care is available to all regardless of income or geography. Approximately 20% of the population hold private health insurance, often employer-funded; interventions described here do not target privately insured hospital care. Our experience may not be directly applicable to countries with different health-care organisation.

## Data Availability

No data are available.

## Declarations

### Climate effect

Following the intervention, the climate effect of the interventions will be evaluated, based on estimated reductions in CO₂ emissions from patient travel and procedural volumes.

### Disclaimer

Data from NORIC have been used in this publication. The interpretation and reporting of these data are the sole responsibility of the authors; no endorsement by NORIC is intended or should be inferred.
